# IR-laser assisted additive freeform optics manufacturing

**DOI:** 10.1038/s41598-017-07446-8

**Published:** 2017-08-02

**Authors:** Zhihan Hong, Rongguang Liang

**Affiliations:** College of Optical Sciences, University of Arizona, 1630 E University Blvd, Tucson, Arizona 85721 USA

## Abstract

Computer-controlled additive manufacturing (AM) processes, also known as three-dimensional (3D) printing, create 3D objects by the successive adding of a material or materials. While there have been tremendous developments in AM, the 3D printing of optics is lagging due to the limits in materials and tight requirements for optical applicaitons. We propose a new precision additive freeform optics manufacturing (AFOM) method using an pulsed infrared (IR) laser. Compared to ultraviolet (UV) curable materials, thermally curable optical silicones have a number of advantages, such as strong UV stability, non-yellowing, and high transmission, making it particularly suitable for optical applications. Pulsed IR laser radiation offers a distinct advantage in processing optical silicones, as the high peak intensity achieved in the focal region allows for curing the material quickly, while the brief duration of the laser-material interaction creates a negligible heat-affected zone.

## Introduction

Freeform optics is a recent, emerging and developing field to meet the increasing demands created by the high performance and ultra-compact optical imaging systems that underpin consumer and medical applications, such as mobile phones, head mounted displays, and ultrathin endoscopes^1–3^. While it offers a number of advantages, fabrication is one of the major issues preventing freeform optics from realizing rapid commercial adoption, with current options either incapable of complex freeform optics or prohibitively expensive and time-consuming. For example, ultraprecision single point diamond turning can produce high quality surface finishes on the order of nanometers with tight form tolerances on the order of micrometers; however it is very time-consuming and the cost is high. Additive manufacturing (AM) will be an ideal method to prototype freeform optics if the surface quality specifications can be realized.

The most common approaches for transparent 3D printing include: 1) the “printoptical” technology by Luxexcel Group B.V.^4^; 2) stereolithography (SLA); 3) multi-jet modeling (MJM); 4) polyjet 3D printing^5^; and 5) two-photon polymerization technique^6^. The curing process used for SLA causes visible layers to remain in the product. MJM uses a jetting technology and a wax support material that is melted away once the product shape is complete. Polyjet printing can work with a wide range of materials to form complex geometries. SLA, MJM, and polyjet 3D printing and their commercial systems, however, were developed to print non-optical, non-transparent components. Although post-processing, like sanding and grit blasting, can increase transparency, the surface roughness still cannot meet the demands of optical applications and, most importantly, the surface shape cannot be controlled^7^. Nanoscribe’s Photonic Professional GT uses multiphoton polymerization with direct laser writing, and was developed for complex photonics structure. Its major problem for printing optical components is the small field of view of the microscope objective, which means only very small optics are realistic^8–11^. For optics larger than few hundred microns, the steps between the fields of view are obvious and unsuitable for imaging applications. Luxexcel’s proprietary “printoptical” technology can 3D print transparent products without visible layering by jetting droplets that are merged before being cured^12^. Each of these commercial systems uses an ultraviolet (UV) curing method, either as a standard UV polymerization or two-photon polymerization process. One limitation of the current UV curing systems is the material, which causes the lens to appear yellowish^13^.

In this paper, we demonstrate a new precision additive freeform optics manufacturing (AFOM) method for printing freeform optics from optical silicones by using an pulsed infrared (IR) laser. Specifically, to cure the optical material we will use an IR laser to locally solidify the optical silicones to control the lens shape accurately.

## Method

Optical silicones, such as Polydimethylsiloxane (PDMS) and Dow Corning® MS-1002 Moldable Silicone, are typically used in LED lighting and other commercial applications^14–17^. Compared to UV curable materials, thermally curable optical silicones have a number of advantages, such as strong UV stability, non-yellowing, and high transmission, making it particularly suitable for optical imaging applications^18^. Hence, lithographic, surface-tension driven, embossing, hanging methods, and a confined sessile drop technique, have been reported to fabricate optics from optical silicones^19–25^. These methods have some common issues: 1) they are limited to simple, small-scale optics; 2) they are slow; and 3) they cannot control the freeform shape to meet design specifications. Although a moving needle method was developed to partially change the lens shape, it too cannot control the lens shape accurately^26^. A printing approach using a passive droplet dispenser has been investigated to fabricate a lens from optical silicone, but the reported method cannot control the lens shape either^27^.

Depending on the silicone chemistry, heat curing can increase temperature resistance, chemical resistance, and improve the strength of the adhesive, especially at elevated temperatures. Getting heat into the optical silicone quickly is key to creating sufficient strength quickly and reducing cycle times. Pulsed laser radiation offers a distinct advantage in processing the optical silicone, as the high peak intensity achieved in the focal region of the objective allows for curing the material, while the brief duration of the laser-material interaction creates a negligible heat-affected zone^28^.

**Figure 1:**
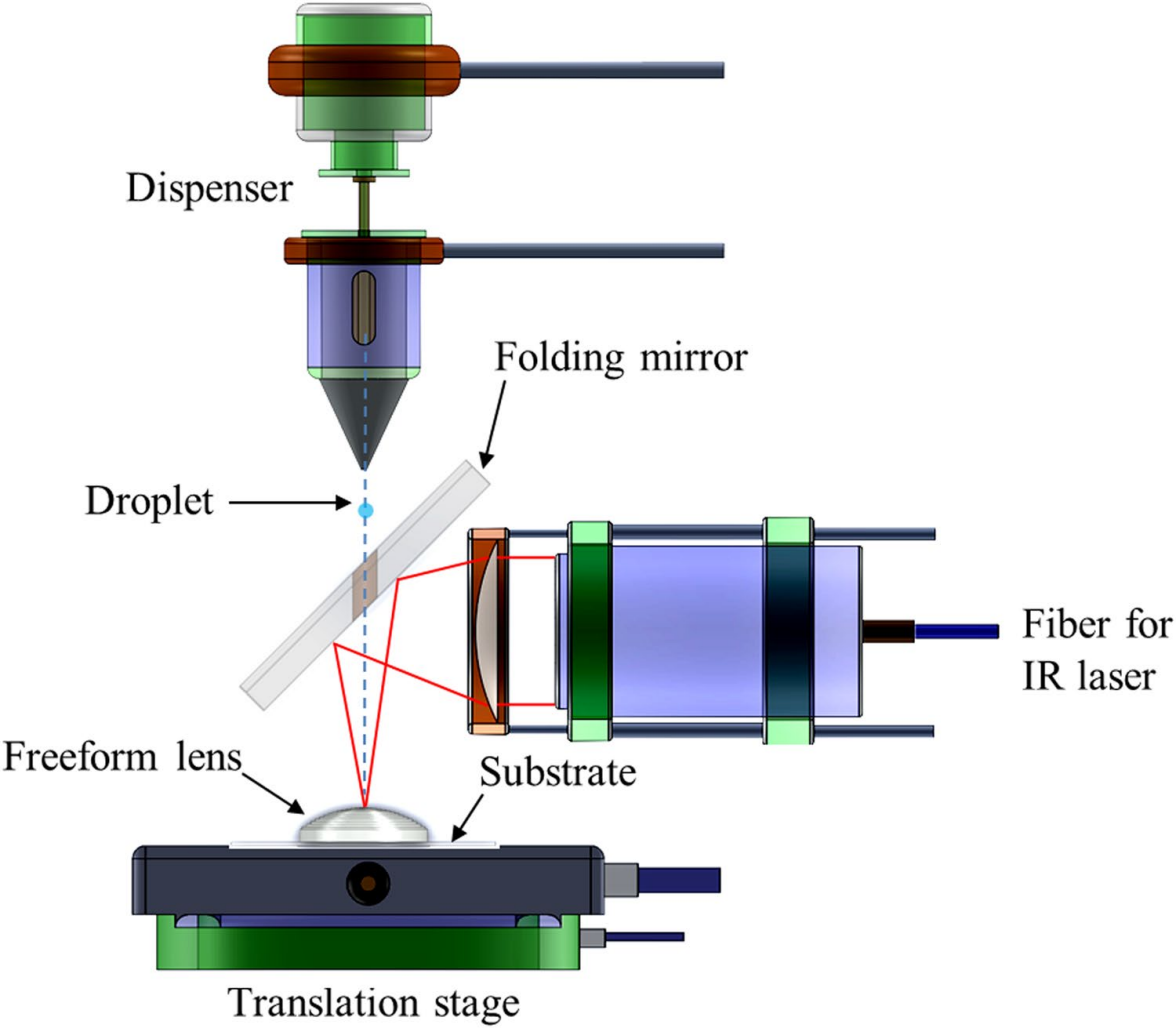
Layout of the AFOM system. The substrate is placed on the one translation stage, the folding mirror, the dispenser, and the laser focusing lens are mounted together in a precision xyz computer-controlled stage.

Figure 1 is the layout of the home-built AFOM system. The glass substrate to hold the printed lens is placed on the translation stage. The material dispenser and the focusing lens are combined by the folding mirror which has a hole in the center, allowing optical silicones reaching the lens substrate. The fiber is used to deliver the the laser light from a Q-switched fiber laser (AP-QS1-MOD, AdValue Photonics Inc) to the focusing lens which has a focal length of 50 mm. The operating wavelength is 1.95 ± 0.05 µm, the average power is 10 W, the pulse repetition rate is 20 kHz, the pulse width 20 ns, the pulse energy is 500 µJ, and the output beam size is 8 mm. The amount of material dispensed from the home-built dispenser is computer controlled, the estimated diameter of the droplet is 200 µm. By mounting the dispenser and focused laser on the translation stage and keeping the lens stationary during the printing process, we will be able to print the lens more accurately because the lens is stationary. Three Thorlabs’s motorized translation stages PT1-Z8 are used in the experiment, the specifications are: min achievable incremental movement is 0.05 µm, min repeatable incremental movement is 0.2 µm, and the resolution is 29 nm. The printing system is also applicable for drop-on-demand printing method. The focused laser spot will heat and cure the tiny droplets on the lens surface.

**Figure 2:**
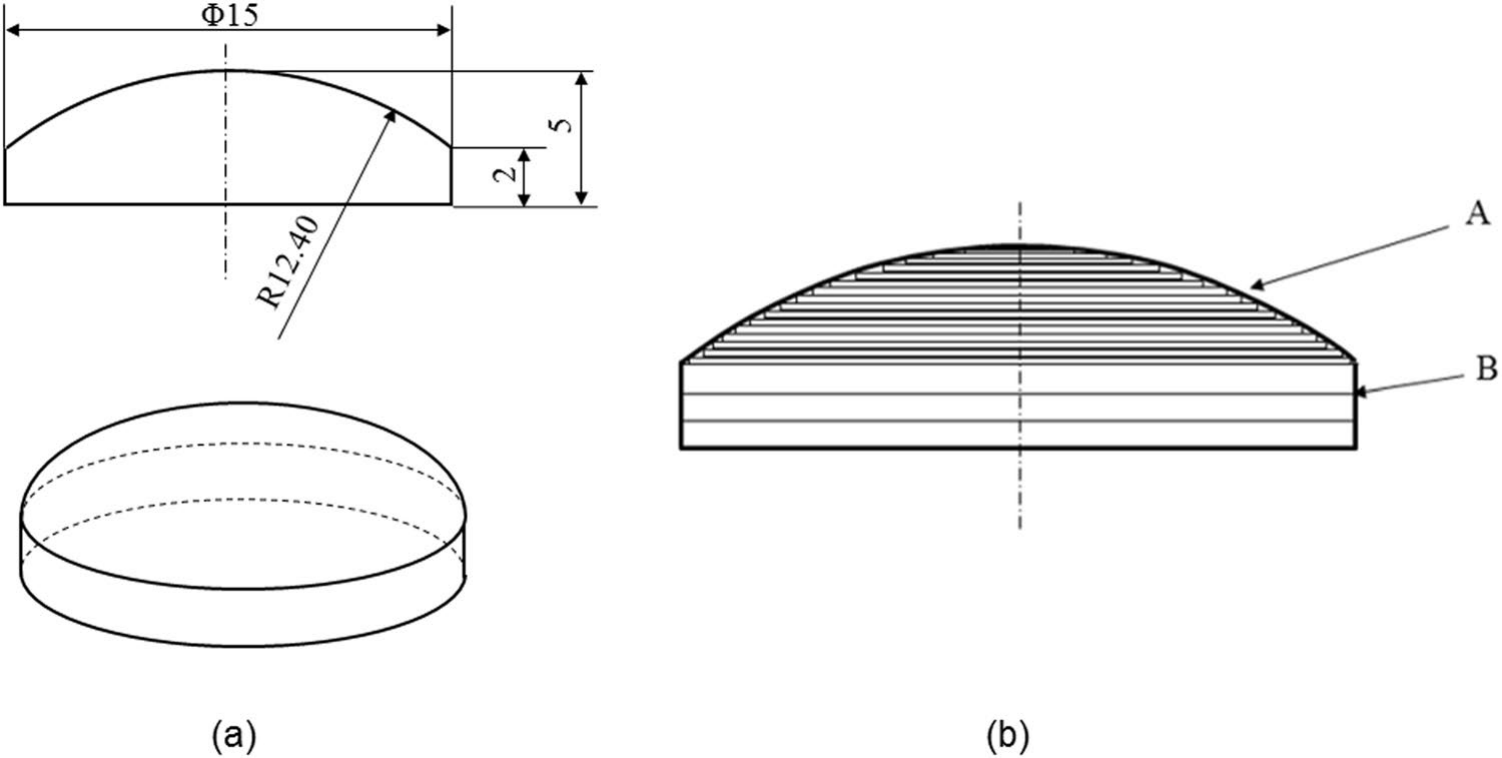
(**a**) The parameters of the plano-convex lens, and (**b**) the schamtic drawoing showing the printing plan. The thin lines show the thickness of each printed layer.

We have developed three different approaches to print freeform optics using optical silicones and IR laser: layer-by-layer, drop-on-demand, and hybrid methods. Drop-on-demand method can control the lens shape easier, but precise control of the droplet size is needed. The lens shape in layer-by-layer method is controlled by the laser spot size, meaning the printing process is more critical. Hybrid method takes the advantages of layer-by-layer and drop-on-demand method, but the system control is more difficult. In this paper, we will only discuss the layer-by-layer method in details. Using the plano-convex lens in Fig. 2(a) as an example, it has a thickness of 5 mm, a radius of 12.4 mm, and a diameter of 15 mm. As the first step, we will need to determine the layer thickness during the printing process with the goals of fast printing and good surface quality. Ideally, for the curved surface A in Fig. 2(b), the thinner the layer thickness, the accurate the surface shape. However it will take more time to print. For the flat surface, for example the section B, the layer thickness can be larger. Figure 2(b) schematically shows the printing layer thickness for the lens in Fig. 2(a). We have developed the slope-based methods to determine the layer thickness with the trade-offs between the printing speed and surface shape. For the reported study in this paper, the layer thickness of the flat surface is 100 µm and the layer thickness for the curved surface is 20 µm, both of them are uniform across the entire lens.

**Figure 3:**
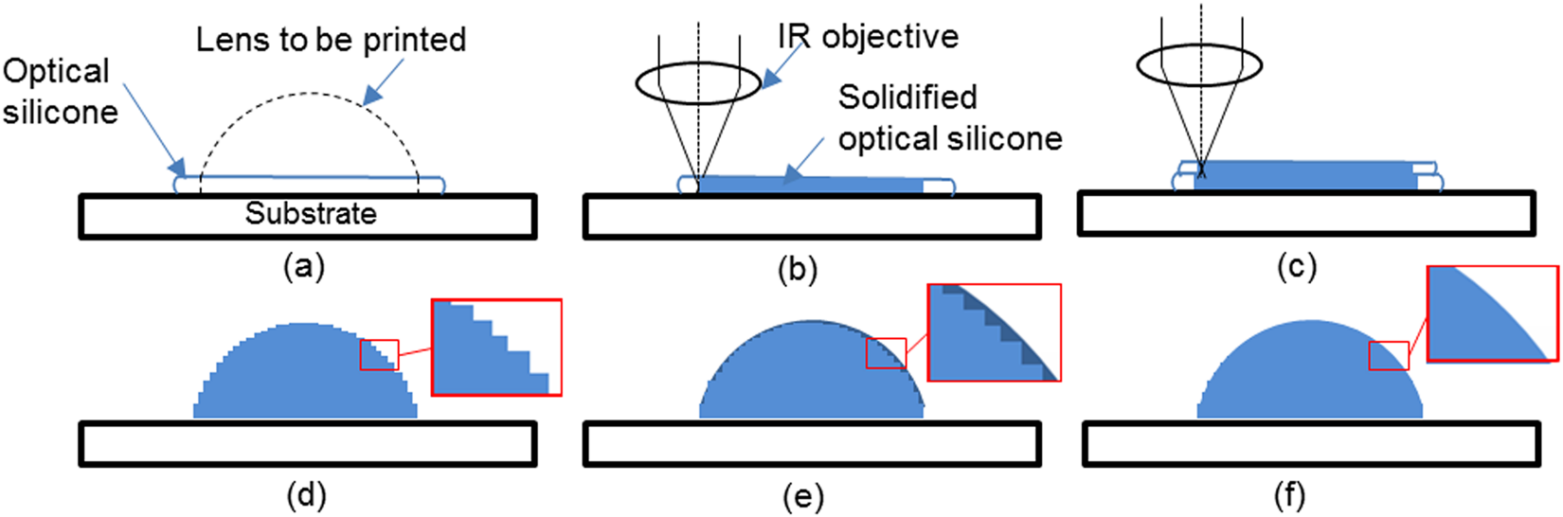
The printing process used in the current study. Lens to be printed is shown in (**a**) to demonstrate the printing process. (**a**) The material is dropped and flows on the substrate, (**b**) the laser is scanned across to cure the material, (**c**) more layers are printed on the top of the cured layer, (**d**) the process is repeated until the lens shape is formed, (**e**) the material is dropped to fill the steps between the layers to smooth the surface, and (**f**) the lens whole surface is cured with laser.

Figure 3 shows the printing processing. As the first step, the material is dropped on the substrate. After the material flows uniformly over the substrate or on the solidified lens surface as shown in Fig. 3(a) or (c), the focused laser beam solidifies the material rapidly to form the shape of each layer (Fig. 3(b) and (c)). By repeating the same steps, we are able to form the lens shape shown in Fig. 3(d). The unsolidified material is accumulated on the substrate and is spun off. Due to the layer-by-layer curing process, steps exist between each layer no matter how thin the layers (Fig. 3(d)). To smooth the lens surface, we apply a relatively large amount of material from the top to fill these steps (between each layer, Fig. 3(e)) and then cure the surface (Fig. 3(f)).

The surface shape and quality are determined by a number of factors, such as the layer thickness in layer-by-layer printing process, the droplet size in drop-on-demand process, laser power, pulse duration, printing speed, and the material properties. For each surface type, there is a need to develop the optimal printing process, and the same is true for each type of material.

## Results

**Figure 4:**
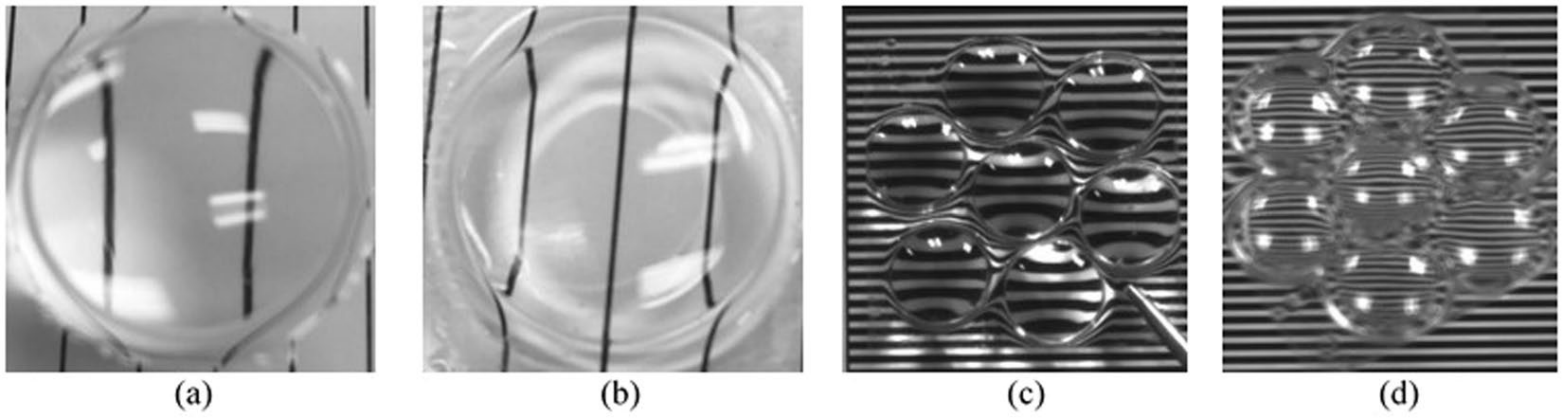
Printed PDMS lenses. (**a**) Plano-convex lens (the diameter = 15 mm), (**b**) plano-concave lens, (**c**) plano-convex lens array, and (**d**) plano-concave lens array.

Figure 4 shows a variety of printed PDMS lenses. Together these lenses demonstrate that the proposed AFOM approach with a pulsed IR laser will not only print simple plano-convex lenses (Fig. 4(a)), but can also thermally print plano-concave lens (Fig. 4(b)) that has not been demonstrated by other thermal curing methods before. The plano-convex lens array and plano-concave lens array in Fig. 4(c) and (d) are two other freeform optics examples showing the range of AFOM. Compared to the yellowish UV curable lenses reported in various publications^13^, the lenses in Fig. 4 are optically clear, not showing any yellowness, much better for imaging applications.

**Figure 5:**
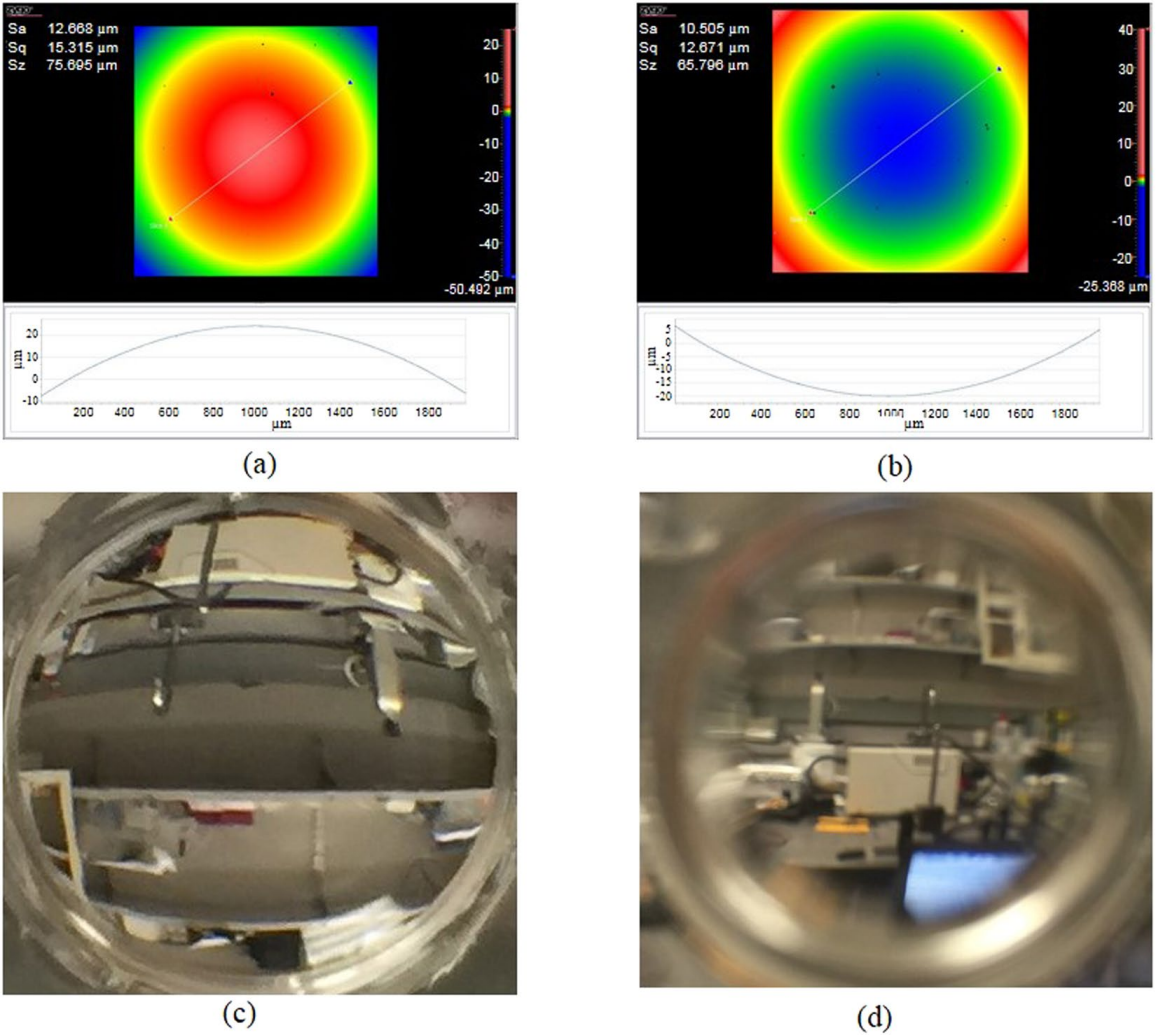
The measured surfaces of the convex and concave lenses in Fig. 4(a) and 4(b) with Zygo Newview 8300 white light interference microscope. (**c**) and (**d**) are the images of the lab obtained with the lenses in (**a**) and (**b**).

Figure 5 are the measured convex and concave surfaces in Fig. 4(a) and 4(b) using Zygo Newview 8300 white light interference microscope. The measured results demonstrate the AFOM is able to print lens surface accurately. The RMS surface roughness is less than 20 nm, which can be improved by reducing the layer thickness and using the focusing lens with larger numerical aperture (NA). The NA of the current focusing lens is 0.08, the diameter of the focused spot is about 30 µm. Figure 5(c) and 5(b) are the images of the lab using the lenses in Fig. 5(a) and 5(b), further demonstrating the imaging capability of the printed lens.

**Figure 6:**
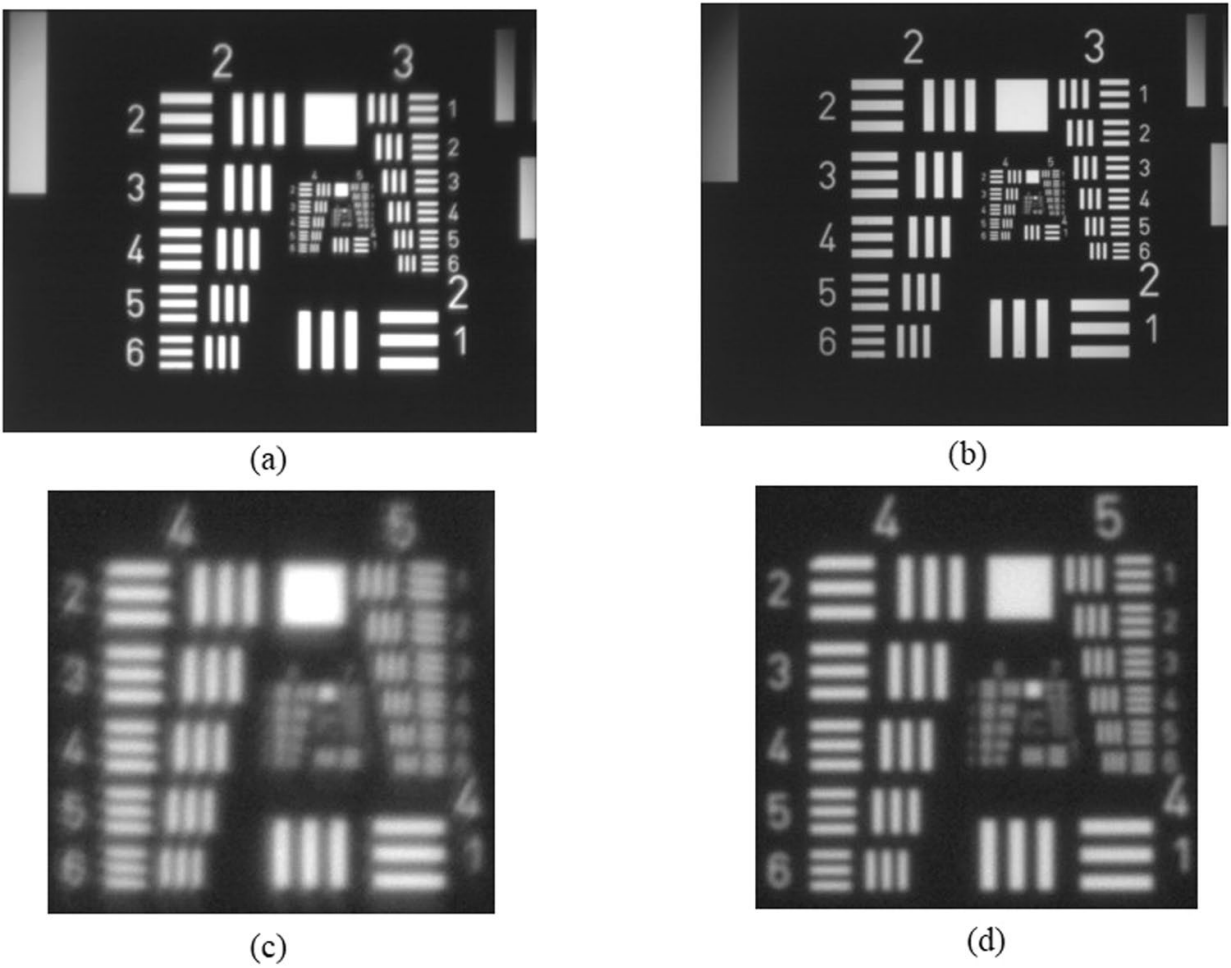
The images of USAF 1951 resolution target with (**a**) printed PDMS lens in Fig. 4(a) and polished glass lens 10.0035 from Rolyn Optics Inc. (**c**) and (**d**) are central regions in (**a**) and (**b**).

To further study the performance of the printed lens, we compare the performance of the printed PDMS lens with the commercial polished glass lens. Figure 6(a) is the image of the USAF 1951 resolution target with the printed lens in Fig. 4(a) and a point-grey camera (FL3-U3-13S2C-CS), Fig. 6(b) is the image captured by a plano-convex glass lens with the radius of 12.533 mm (Lens 10.0035 from Rolyn Optics Inc). Figure 6(c) and 6(d) are the central regions of Fig. 6(a) and 6(b). The study demonstrates that the performance of the printed lens in imaging system is close to the commercial, polished glass lens. The maximum resolution (in the object side) for the printed lens is 36 lp/mm, while it is 45 lp/mm for the polished glass lens. Both the resolution and contrast are slightly lower than that of the polished glass lens. The glass lens should have better imaging performance because (1) its refractive index (1.516) is higher than that of the PDMS (1.41), therefore the spherical aberration is lower, and (2) the surface shape of the polished lens is more accurate than the printed lens used in this experiment.

**Figure 7:**
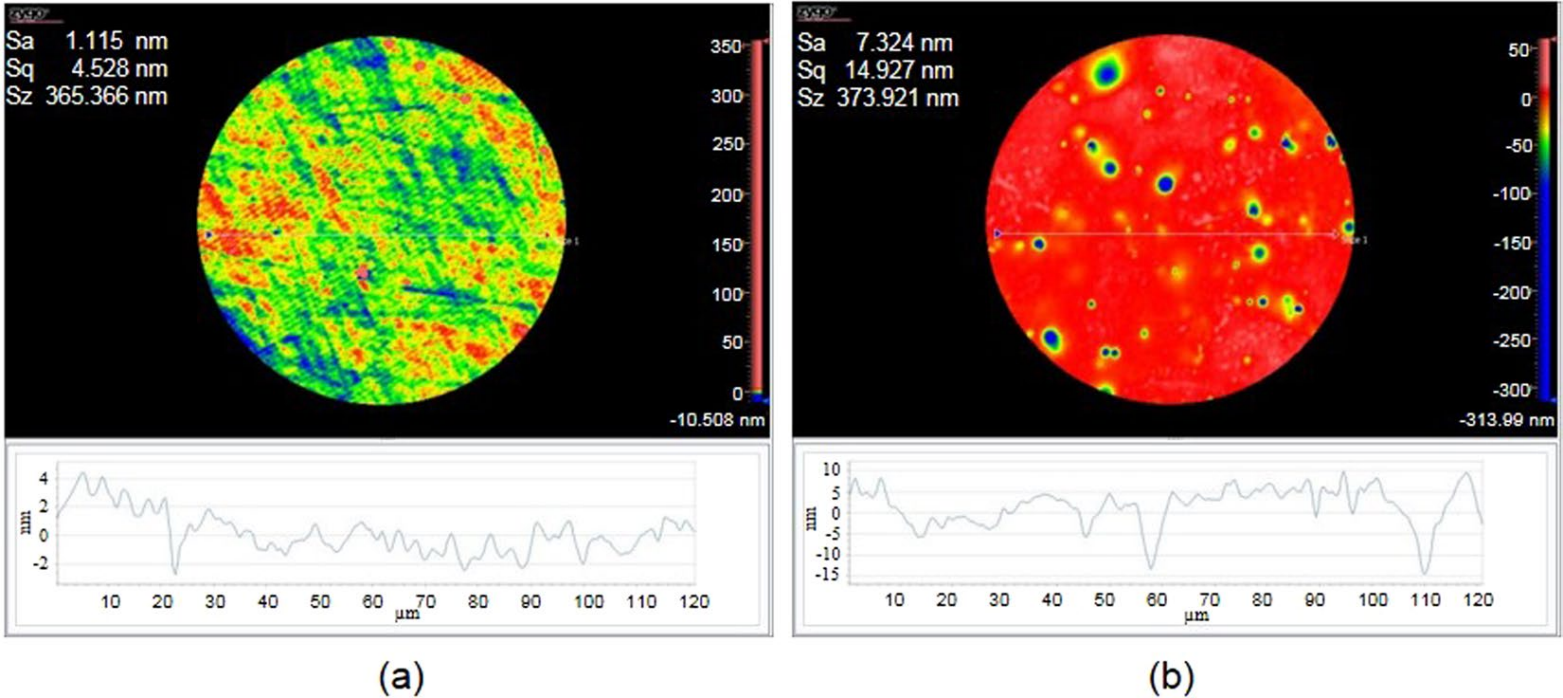
Measured surface roughness. (**a**) Polished glass lens, and (**b**) printed lens in Fig. 5(a).

The lower contrast of the image captured by the printed lens is mainly due to the fact that the surface roughness of the printed PDMS lens is larger than the polished glass lens. As a comparison, Fig. 7 shows the measured surface roughness of the polished lens and the printed lens. The RMS surface roughness of the polished lens is less than 5 nm, while it is about 15 nm for the printed lens. The peaks in Fig. 7(b) could be due to the dusts which accumulate on the lens surface during the printing process.

**Figure 8:**
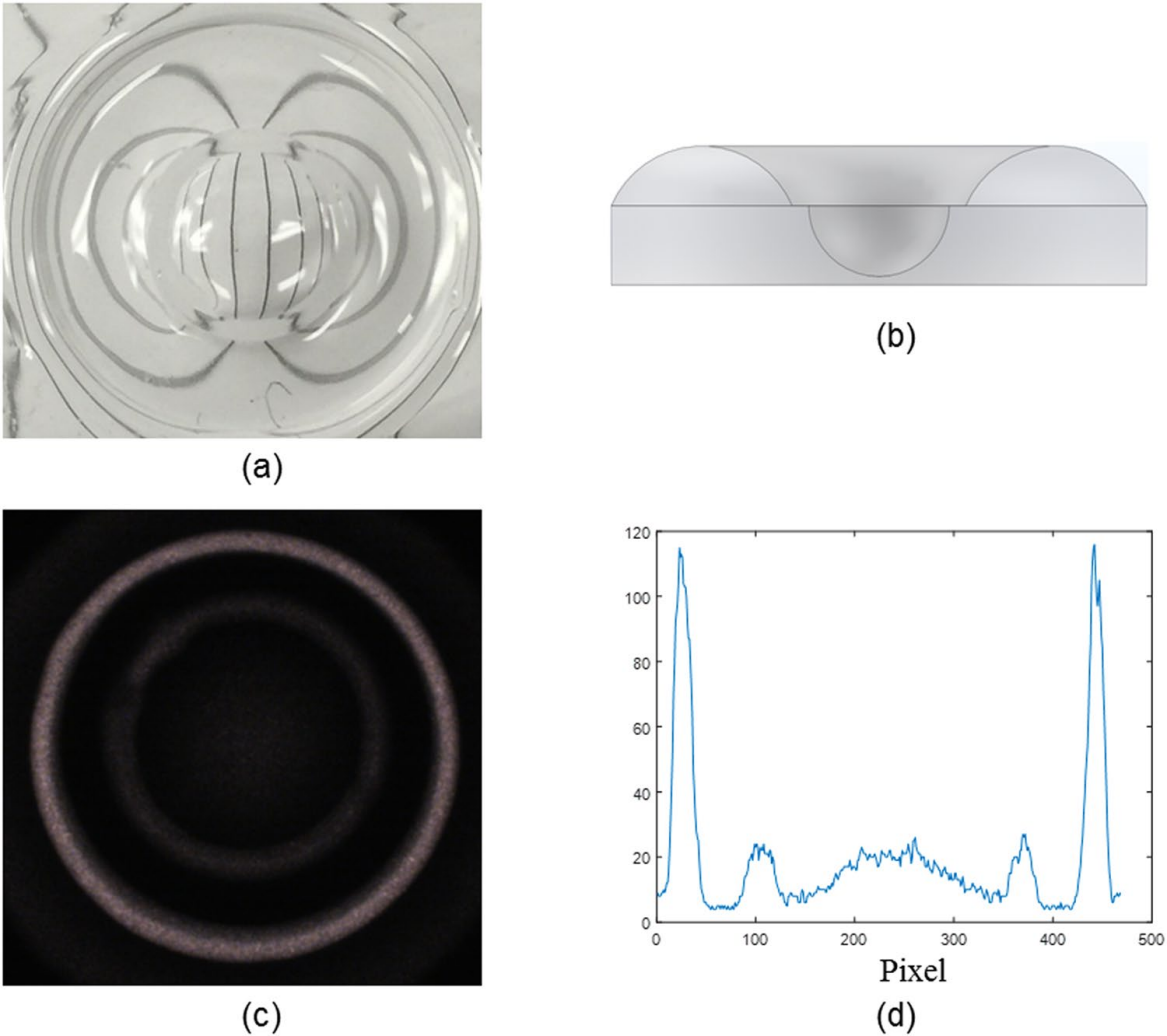
Example of the printed freeform donut lens using AFOM process. (**a**) Printed donut lens, (**b**) lens cross-section, (**c**) the focused pattern of the donut lens with fiber light source, and (**d**) the profile of the focused pattern.

To further demonstrate the capability of the proposed AFOM process, we print a freeform donut lens in Fig. 8(a), its cross-section is shown in Fig. 8(b). The lens diameter is 16 mm and the thickness is 4 mm. This donut lens can focus the light to a ring pattern as shown in Fig. 8(c), the horizontal profile is plotted in Fig. 8(d). The illumination profile is correlated to the lens structure directly, the donut lens focuses the light from the light source to a strong outer ring, the weaker inner ring is due to the flat transition region in the donut lens. The donut lens in Fig. 8(a) is another freeform optics that leads credence to the fully developed AFOM method being able to control the freeform lens shape.

## Conclusion and Discussion

For the first time we have developed the pulsed IR-laser assisted additive manufacturing process to print freeform optics from optical silicones. We have demonstrated the feasibility and capability of the proposed AFOM methods, the key advantages include (1) the printed optical elements from optical silicones have much better transmission compared to the UV cured optical elements and (2) the AFOM process with the pulsed IR laser can print freeform lenses accurately.

In order to print the lens more accurately, we will further develop the system by using dispenser which can dispense smaller amount of the material and the focusing lens with large NA. In addition, we will incorporate the *in-situ* metrology capability into the system. *In situ* metrology will measure the cured surface in real time and provide feedback to the printing process. If there is deviation from the predefined intermediate lens shape, the printing process will be fine-tuned to compensate the deviation.
